# Immediate breast reconstruction following segmentectomy using a latissimus dorsi ‘myoadipose’ flap through a single axillary incision: a case series

**DOI:** 10.4076/1757-1626-2-8116

**Published:** 2009-06-11

**Authors:** Vijay Naraynsingh, Seetharaman Hariharan, Dilip Dan

**Affiliations:** Department of Clinical Surgical Sciences, Faculty of Medical Sciences, The University of the West IndiesSt. Augustine, TrinidadWest Indies

## Abstract

Immediate breast reconstruction is an excellent complementary treatment for patients with ductal carcinoma in situ and early-stage invasive breast cancer. Although lattisimus dorsi *myocutaneous* flap is one of the well accepted and versatile methods of breast reconstruction, there have been very few reports describing a *myoadipose* flap, especially through the same axillary incision used for nodal clearance. This article describes such a technique which produced excellent results both surgically as well as cosmetically.

## Introduction

Latissimus dorsi myocutaneous flaps are commonly reported to be one of the most versatile and well accepted methods of breast reconstruction after surgery for cancer [1]. There was a report of using autologous fat tissue along with the muscle flap for augmentation mammoplasty as early as 1995 [2]. However, most previous reports described reconstruction following mastectomy. There have been a few recent reports using a rolled lattisimus dorsi muscle flap, to fill segmentectomy defects [3,4]. We report our experience with using lattisimus dorsi myoadipose flap raised through an axillary incision to fill segmentectomy defects. This technique produced excellent results both surgically as well as cosmetically.

### Technique

**Figure 1.**
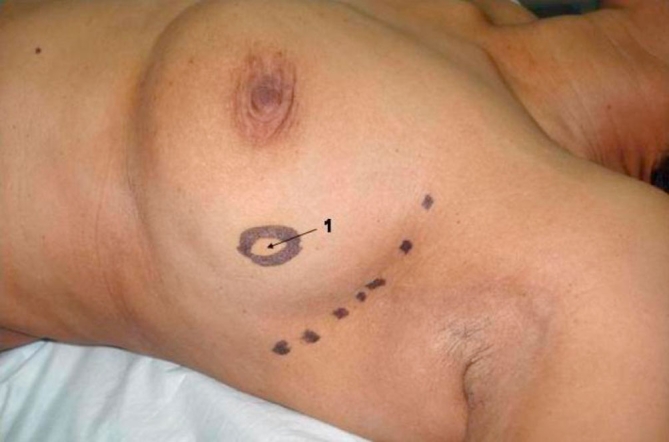
Curvilinear axillary incision.
3 cm tumor in the upper quadrant.

**Figure 2.**
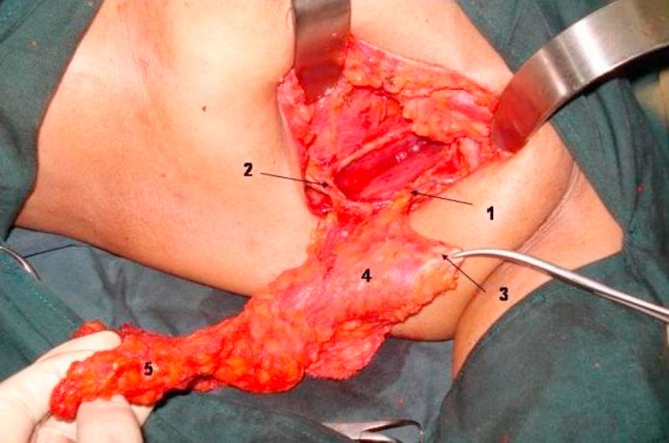
Myoadipose flap with details.
Thoracodorsal pedicle.Communicating branch to the lateral thoracic vessel.Tendon severed from humerus to increase mobility to the flap.Proximal part of muscle tissue devoid of fat.Dissection of axillary fat overlying the flap.

Through a curved 5-7 cm axillary incision from the anterior to posterior axillary lines a level II axillary clearance is done (Figure 1). This incision is adequate for all lateral lesions of the breast. A separate circumareolar incision may be needed for superomedial lesions. We have not used this flap for inferomedial defects. Careful dissection is performed to preserve the thoracodorsal neurovascular bundle. The collaterals between the branches of the thoracodorsal and the lateral thoracic vessels are preserved, thus providing a dual blood supply to the flap (Figure 2). The plane between latissimus dorsi postero-laterally and serratus anterior and the chest wall antero-medially is developed. Muscle trauma is minimized by sharp dissection. Dorsally, much of the subcutaneous fat is left attached to the underlying muscle by diathermy cutting between the skin and fat, leaving only a small amount of fat attached to the skin. A considerable length of muscle with overlying fat could be mobilized by downward traction of the postero-inferior edge of the axillary incision and upward traction of the latissimus dorsi. The fully mobilized muscle along with the pad of fat is divided inferiorly and medially. While this division leaves some of the inferior and medial origins of the muscle, enough of it can be retrieved with the overlying fat, to fill any segmentectomy defect. It is then rotated anteriorly and tacked into position to fill the defect in the breast tissue, caused by the segmentectomy.

**Figure 3.**
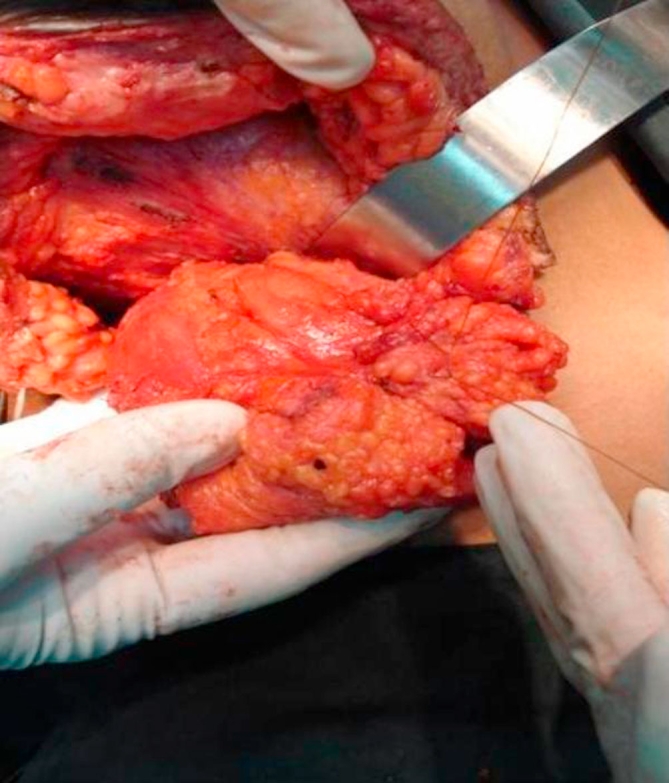
Flap rolled with loose sutures to provide bulk.

**Figure 4.**
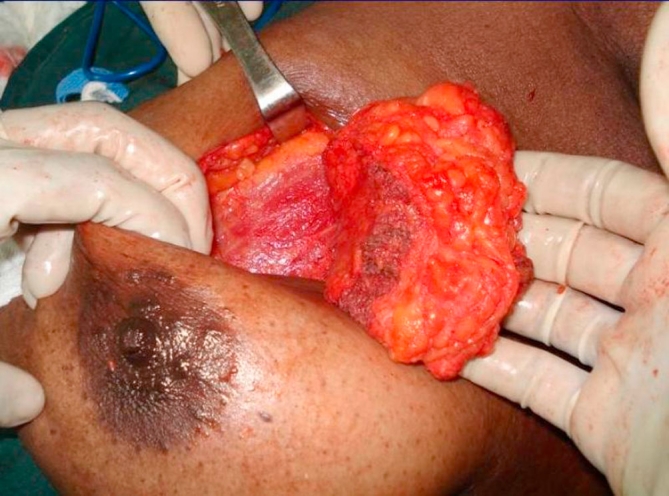
Pad of fat making the flap four times thicker than muscle alone.

The tendon of lattisimus dorsi should be divided from its humeral attachment so that the myoadipose flap can swing into the breast defect without tension (Figure 2). Only muscle, without any attached fat, is mobilized at the humeral end of latissimus dorsi; this eliminates the potential bulge in the axilla and maintains breast contour (Figures 2 and 3). Then, an appropriate quantum of the fat overlying the distal muscle is utilized to fill the tissue defect and provide a normal contour and symmetry with the contralateral side. This can be done by comparing the size of the breast tissue removed and the fat overlying the muscle. If the defect is huge and the myoadipose flap thin, it can be made more bulky by rolling its tip with loose sutures (Figure 4). A suction drain is placed in the axilla and removed after about two weeks. Postoperatively, the patient is advised to move the arm liberally, facilitated by adequate intravenous and oral analgesia.

## Case presentation

We have done a total of five cases by the above technique with similar results (Table 1); the cases described here document flap features in obese and thin patients with large and small breasts and cancerous lump in different quadrants.

### Case report 1

**Figure 5.**
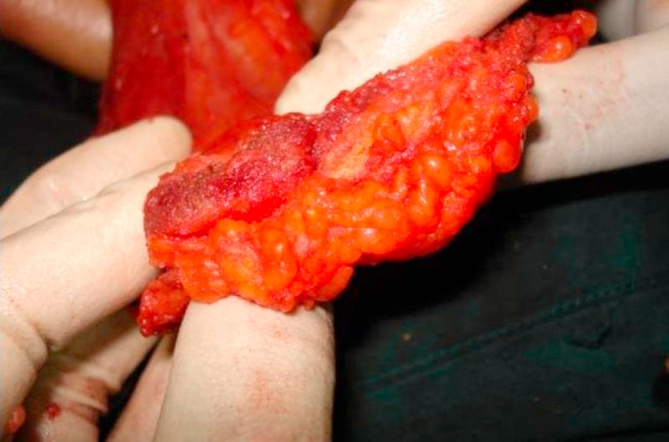
Tip of flap to be rolled in a thin patient.

A 34-year-old Trinidadian female of African ethnicity presented with a 4 cm left breast mass, 4 cm superior to the nipple, confirmed to be carcinoma on cytology. She requested breast conservation and was advised to have a lattisimus dorsi myoadipose flap since the breast deformity would otherwise be significant. A single curved incision near the axilla permitted very wide local excision of the mass as well as adequate mobilization of the myoadipose flap. Since she was moderately obese the fat pad overlying lattismus dorsi quadrupled the flap thickness thus providing generous bulk to fill the breast defect (Figure 5). Histology confirmed 1 cm tumour free margins with 3 of 17 axillary lymph nodes being positive.

**Table 1.** Demographic and other variables

Case	Age (y)	BMI	Cup Size	Lump size (cm)	Nodes involved
1	34	43	38 D	4	3/17
2	55	33	34 C	3	0/18
3	37	41	36 B	2	0/13
4	47	34	34 C	2	0/14
5	45	36	34 B	1.5	1/14

### Case report 2

**Figure 6.**
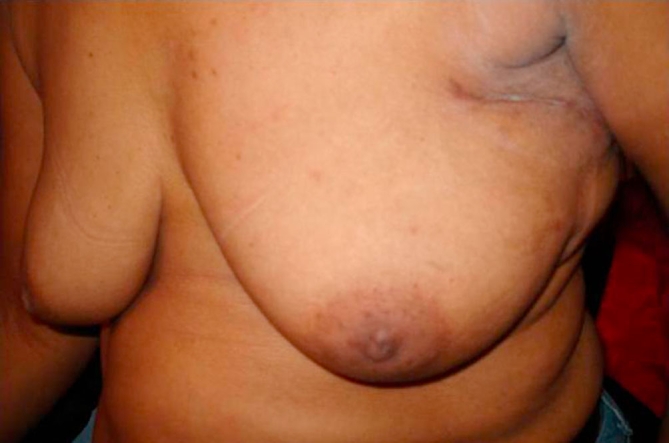
Single scar concealed within the axilla.

A 55-year-old Trinidadian female of African ethnicity presented with a 3 cm cancerous lump in the upper outer quadrant of the left breast. Through a curved axillary incision a very wide excision of the mass was done. The resulting defect was filled with a lattisimus dorsi myoadipose flap. The top of the flap was rolled to increase its bulk as the defect was quite large and the patient did not have a great deal of subcutaneous fat (Figure 6). Histology confirmed 1 cm tumour free margins with no involvement of 18 axillary lymph nodes.

### Case report 3

**Figure 7.**
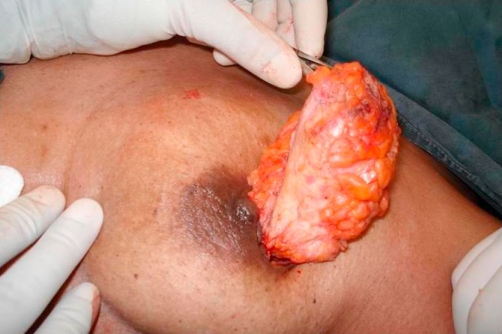
Excision of the upper inner quadrant lesion by a circum-areolar incision.

**Figure 8.**
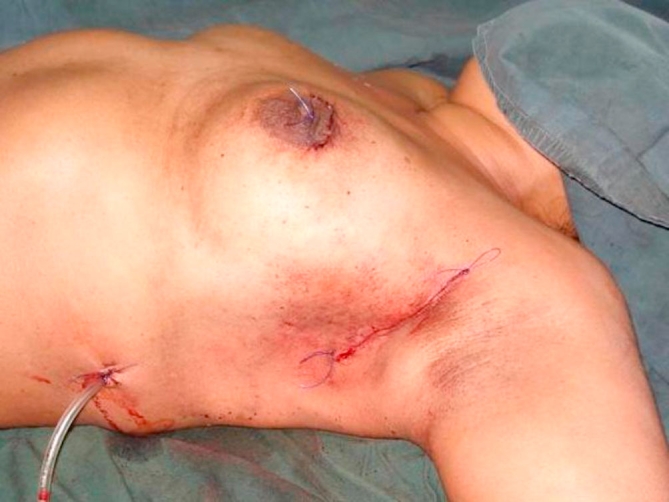
Circum-areolar and axillary incisions with restoration of the breast contour for upper inner quadrant lesions.

A 37-year-old Trinidadian female of Asian Indian ethnicity had FNAC confirmed 2 cm carcinoma located in the upper inner quadrant 1 cm supero-medial to the areola. Axillary clearance and raising the lattissimus dorsi myoadipose flap were done through the same incision as described in the ‘technique’ section. To achieve adequate clearance of the primary lesion, a circum-areolar incision was used (Figure 7). Since the tendon of the flap was divided, it was easy to rotate it into the defect thus restoring the breast contour (Figure 8). Histology confirmed 1 cm tumour free margins with uninvolved 13 axillary lymph nodes.

### Case report 4

A 47-year-old Trinidadian female of Asian Indian ethnicity had FNAC confirmed 2 cm carcinoma located 2 cm superior to the left areola with no axillary lymphadenopathy. A wide local excision of the mass was done through the same incision used for mobilizing the lattisimus dorsi myoadipose flap. Axillary clearance produced 14 nodes, all free of tumour with histologic confirmation of 1 cm negative margins of the primary lesion.

### Case report 5

A 32-year-old Trinidadian female of African ethnicity had FNAC confirmed 1.5 cm carcinomatous lump in the left breast located 2 cm inferolateral to the areolar margin. This was excised through the axillary incision; axillary node clearance and lattisimus dorsi myoadipose flap mobilization were done through the same incision. Histology confirmed 1 cm negative margins of the primary lesion and one axillary node of the 14 retrieved was involved.

All cases are routinely referred to the medical oncology team for adjunctive therapy as they see fit. Patients have been followed up for 6-43 months (mean 14 months) with no evidence of local recurrence.

## Discussion

Immediate breast reconstruction is an excellent complementary treatment for patients with ductal carcinoma in situ and early-stage invasive breast cancer [5]. The latissimus dorsi myocutaneous flap with prosthesis has been considered to be an effective and aesthetic method of immediate breast reconstruction following skin-sparing mastectomy [6,7]. There have also been reports of refinements to the latissimus dorsi flaps which include harvesting subcutaneous fat to avoid implants; however, these were following mastectomy [8-10]. Although wide local excision can produce significant deformity of the breast, little has been reported on using lattisimus dorsi to restore breast contour in these cases. Latissimus dorsi flaps are usually raised using a separate incision in the back and tunneling the pedicled flap through the axilla to the front for reconstruction. The major disadvantages to this technique are a separate, long scar in the back, a need to change the position of the patient for the posterior dissection and a contour defect in the back [11]. Avoidance of a posterior scar has been described in skin sparing mastectomy [9,10]. There are also previous reports recommending the use of single axillary incision for reconstruction following quandrantectomy, although an additional pad of fat was not utilized [3,4]. One of the methods to minimize the scar may be endoscopically harvesting the latissimus dorsi, although a previous report found no additional advantage to this technique [12].

Our modification of the technique offers the advantage of a single incision for axillary clearance as well as harvesting the fat with the muscle flap. Sometimes, depending on the site of the primary it is possible to also do the segmentectomy through the same incision. The scar does not extend posteriorly and is completely concealed with the arm in the normal anatomical position (Figure 3). Additionally, a thick pad of fat along with the muscle can triple or quadruple the bulk for filling the breast defect (Figure 5). This permits generous, uncompromised wide clearance of the tumor with minimal or no deformity. Although there is no scar posteriorly, there is a visible depression in the area from which muscle and fat have been removed. Since the lattissimus dorsi myoadipose flap has a dual blood supply it is quite robust and can tolerate the usual radiotherapy which follows wide local excision of a breast carcinoma. Denewer *et al*. have been able to further increase the bulk of the flap by adding part of serratus anterior and overlying fat to fill mastectomy defects [10]. This would be of value for large defects such as those produced by nipple-sparing mastectomy.

Thus far, we have used this flap to fill defects in any portion of the breast except the infero-medial quadrant. For supero-medial lesions, an additional circumareolar incision is used to provide adequate clearance of the primary lesion (as in our case 3). A recent study reported that immediate reconstruction following partial mastectomy using lattisimus dorsi flaps had a higher complication rate than using local tissue [13]. However, we believe that increasing the vascularity by preserving the dual blood supply, improving its mobility by division of the lattisimus dorsi tendon and augmenting its bulk by utilization of the overlying fat would significantly minimize the complications associated with this procedure.

Satisfactory oncologic outcomes have been assessed by histologic margins of the primary lesion and the number of axillary nodes retrieved. Much longer follow up would be needed to assess the overall oncologic outcomes.

In summary, immediate breast reconstruction following segmentectomy, by raising a latissimus dorsi myoadipose flap from the same incision used for axillary clearance offers excellent cosmetic and oncologic results.

## Abbreviation

FNAC: Fine-Needle Aspiration Cytology
